# A Deep-Learning-Driven Light-Weight Phishing Detection Sensor

**DOI:** 10.3390/s19194258

**Published:** 2019-09-30

**Authors:** Bo Wei, Rebeen Ali Hamad, Longzhi Yang, Xuan He, Hao Wang, Bin Gao, Wai Lok Woo

**Affiliations:** 1 Department of Computer and Information Sciences, Northumbria University, Newcastle upon Tyne NE1 8ST, UK; rebeen.hamad@northumbria.ac.uk (R.A.H.); longzhi.yang@northumbria.ac.uk (L.Y.); wai.l.woo@northumbria.ac.uk (W.L.W.); 2 School of Sino-Dutch Biomedical & Information Engineering, Northeastern University, Shenyang 110169, China; hexuan@bmie.neu.edu.cn; 3 Neusoft Research of Intelligent Healthcare Technology, Co. Ltd., Shenyang 110169, China; 4 Automation College, Chongqing University of Posts and Telecommunications, Chongqing 400065, China; wanghao@cqupt.edu.cn; 5 School of Automation Engineering, University of Electronic Science and Technology of China, Chengdu 610054, China; bin_gao@uestc.edu.cn

**Keywords:** phishing detection, cyber security, deep learning

## Abstract

This paper designs an accurate and low-cost phishing detection sensor by exploring deep learning techniques. Phishing is a very common social engineering technique. The attackers try to deceive online users by mimicking a uniform resource locator (URL) and a webpage. Traditionally, phishing detection is largely based on manual reports from users. Machine learning techniques have recently been introduced for phishing detection. With the recent rapid development of deep learning techniques, many deep-learning-based recognition methods have also been explored to improve classification performance. This paper proposes a light-weight deep learning algorithm to detect the malicious URLs and enable a real-time and energy-saving phishing detection sensor. Experimental tests and comparisons have been conducted to verify the efficacy of the proposed method. According to the experiments, the true detection rate has been improved. This paper has also verified that the proposed method can run in an energy-saving embedded single board computer in real-time.

## 1. Introduction

**Figure 1:**
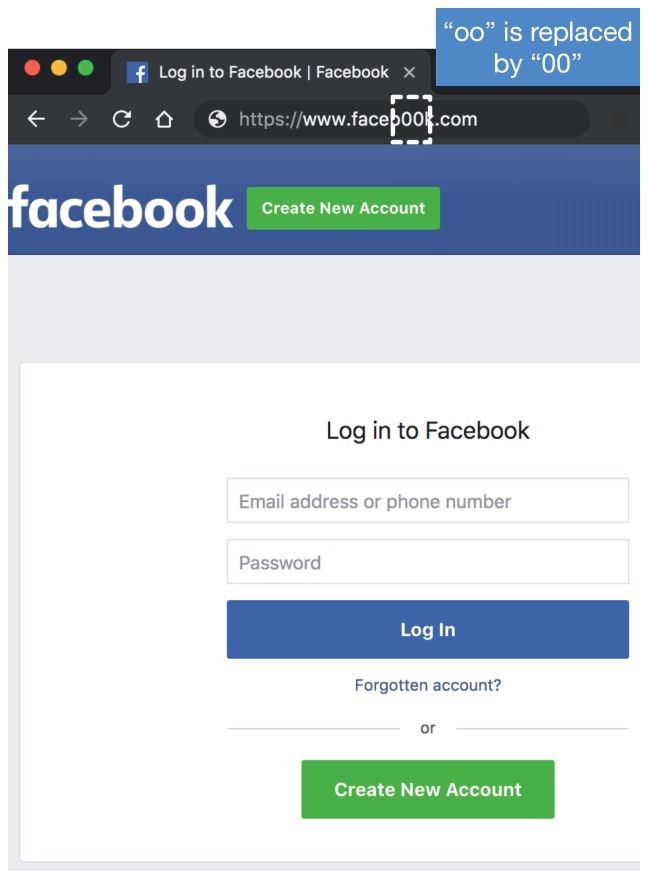
One example of a phishing website imitating the popular website facebook.com.

A phishing website is a common social engineering method that mimics trustful uniform resource locators (URLs) and webpages to gather users’ sensitive and confidential information, such as user names, passwords, credit card information, etc. Figure 1 shows one example of a phishing website imitating the popular website facebook.com. It replaces “oo” with the unnoticeable “00”. The webpage looks exactly the same as the official Facebook, but the phishing one will keep the username and passwords of victims and forward them to attackers. The phishing website issue is becoming increasingly severe. According to the latest phishing activity trends report from the Anti-Phishing Working Group (APWG) [1], 138,328 phishing websites were reported in the fourth quarter of 2018. The report also indicates the increasing trend of detection difficulty because attackers are trying to use multiple redirection techniques in order to make the malicious URLs obscure. There was a $48-million financial loss due to phishing in the US in 2018, only based on the cases reported to the Federal Bureau of Investigation (FBI) [2].

**Figure 2:**
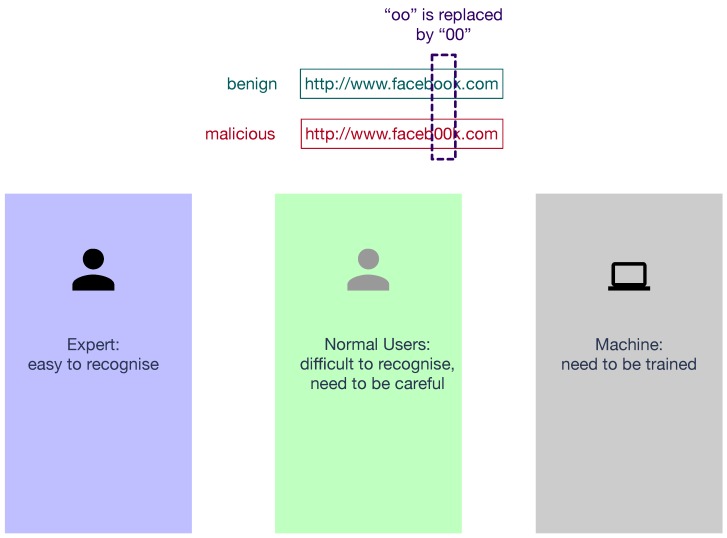
Difficulties to recognise malicious URLs for experts, normal users, and machines.

As shown in Figure 2, malicious URL recognition is relatively easy for cyber security experts since they have sufficient experience in the relevant areas. However, it is extremely difficult for normal users who usually do not pay much attention when accessing one URL. Therefore, the research community takes advantage of the expert knowledge of cyber security and designs machine-based automatic phishing URL detection. The most popular method to detect a phishing website is the use of a phishing URL tank. The URLs in that tank will be recognised as phishing URLs. Phishing URL tanks are maintained by antiphishing organisations to provide live antiphishing databases. There are several famous antiphishing organisations, such as phishtank [3], Joewein [4], hphosts [5], Malware Domains List [6], etc. Due to the rapidly increasing number of phishing websites, antiphishing organisations require comprehensive contributions from the whole community. To maintain up-to-date phishing URL tanks, they need users, including individuals and organisations, to report phishing websites manually. The URLs are fairly accurate because of this manual involvement, but there are still drawbacks in that the human effort introduces delay and extra maintenance labour costs. These handcraft list-based phishing website detection could effectively prevent further harm, but this may fail to promote warnings before its URL is reported by one user and placed in the phishing tank.

**Figure 3:**
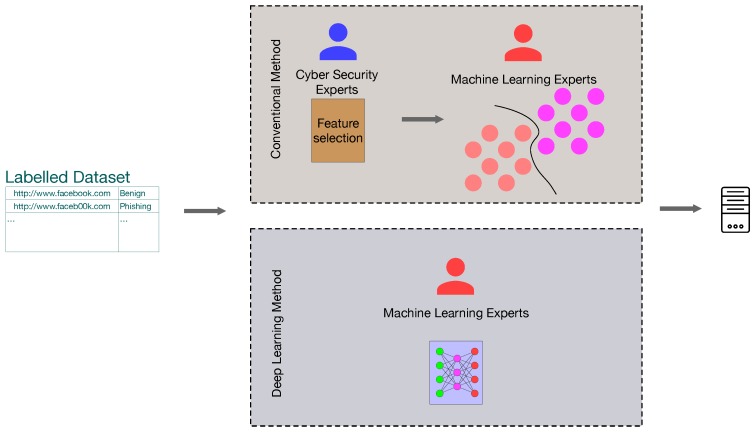
The phishing detection methods using classical machine learning methods and deep learning techniques.

Conventional machine learning techniques have been introduced to the phishing website detection domain [7,8]. As shown in Figure 3, with the help of experts in cyber security, URLs and websites are first analysed to conduct feature selection from the malicious websites. Next, machine learning experts use the features, along with their labels, to construct a training set and take advantage of classical supervised machine learning algorithms to develop a phishing detection model. Many conventional methods, e.g., support vector machine (SVM), k-nearest neighbours algorithm (kNN), etc., have been explored to fully utilise these features. Deep learning is also incorporated in the phishing detection domain, motivated by its recent rapid development and many successful applications [9,10]. Different from classical machine learning methods involving an explicit handcrafted feature selection process, the machine learning experts can use the data directly without the knowledge from the cyber security experts (shown in Figure 3).

This paper designs and implements a light-weight phishing detection sensor. The paper proposes an innovative deep learning model to enable accurate and efficient phishing detection using URLs of websites. Different from previous research works, this research also investigates the feasibility of our proposed deep learning model in resource-constrained computing devices. Furthermore, this research work implements a prototype of a deep-learning-driven light-weight phishing detection sensor in one embedded single board computer, which shows the feasibility of the integration of our method into one wireless router. This work also uses a large volume of benign and malicious URLs to construct the training set and evaluate the efficacy of the proposed model.

In summary, the main contributions of this paper are:

- This research paper proposes a novel character-level multi-spatial deep learning model to detect malicious URLs. The popular convolutional neural networks (CNN) have been explored to improve detection performance.
- This research paper also integrates the proposed model in one single board computer to enable an energy-saving and efficient phishing website sensor. As far as is known, this paper is the first to discuss the feasibility of the usage of resource-constrained computing devices to enable a phishing website detection sensor.
- This paper has conducted extensive evaluations to show the performance of the proposed method and the efficiency of our prototype.

This paper first introduces related work in Section 2. Section 3 shows the background and motivations of the proposed method. The proposed method is introduced in Section 4, and this paper evaluates the performance of the proposed model in Section 5. Section 6 shows the details of the implementation. Finally, Section 7 concludes our work.

## 2. Related Works

The common method to detect phishing websites is the use of blacklists to include all the reported URLs of phishing websites. This method requires largely manual efforts from the whole community. As introduced in Section 1, the blacklists are mainly maintained by antiphishing organisations. Some popular antiphishing organisations are phishtank [3], Joewein [4], hphosts [5], Malware Domains List [6], etc. Whitelists can also be created to exclude the websites that users trust. Some methods are also proposed, aiming to facilitate the labelling process for list-based phishing website detection. Cao et al. proposed a method to automatically update the whitelist from the users’ familiar websites [11]. Jain et al. designed a hyperlink-based phishing detection mechanism to update the whitelist [12]. Sharifi et al. took advantage of search engines to evaluate the legitimacy of websites and create an up-to-date blacklist accordingly [13].

Machine learning has been extensively used in the phishing detection domain. Phishing detection can be classified as a supervised machine learning problem. A large number of phishing websites on blacklists can be analysed and researched by the cyber security and machine learning community. Features from two main components of a website are commonly used for phishing detection. At first, the attackers usually imitate legitimate URLs to lure users into entering phishing websites, so researchers have focused on the analysis of URL for phishing detection. Additional to URLs, the documents implemented to display one website, such as HTML, CSS, and Javascript documents, are also explored for phishing detection. Amrutkar et al. use multiple features from HTML, CSS, and javascript documents from websites to detect the phishing contents [7]. That work also investigates the website features from smart phones and aims to realise real-time malicious website detection on mobile devices. Rule-based features from URLs were explored to detect phishing internet banking webpages [14]. Natural language processing techniques are also explored in [15] to determine a malicious URL, and the authors use seven traditional classifiers along with selected features from URLs to enable an antiphishing system. Zhang et al. [16] and Xiang et al. [8] proposed Cantita and its augmented version Cantita+, which also extracted features from the contents of websites and used multiple machine learning algorithms.

Recently, deep-learning-based methods have been introduced in the phishing website detection domain. Jiang et al. used convolutional neural network (CNN) techniques, a popular model in deep learning, to detect malicious URLs [17]. One deep learning model using word embedding and CNN has also been proposed to detect malicious URLs, file paths, and registry keys [9]. Le et al. proposed URLNet to use CNN for analysing both word-level features and character-level features for malicious URL detection [10]. Yang et al. applied multiple features for detecting phishing URLs [18]. The deep learning technique has also been introduced into phishing email detection [19].

Different from the previous works, this paper proposes a new deep learning model and further investigates the feasibility of enabling an energy-saving phishing website sensor with the integration of the deep learning model in a resource-constrained computing device.

## 3. Background and Motivations

**Figure 4:**
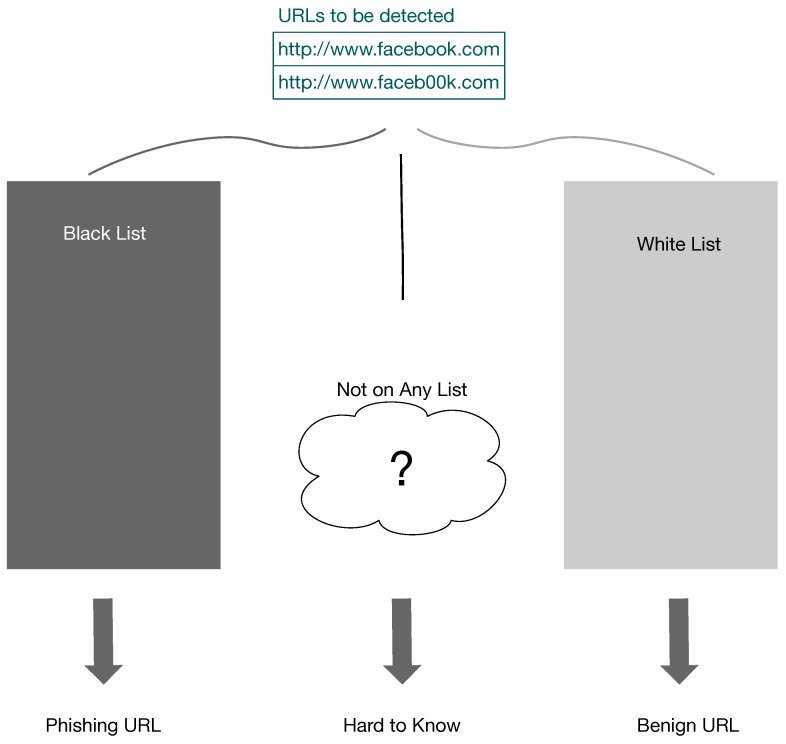
The mechanism of list-based phishing URL detection methods.

List-based phishing website detection is the most common method currently. The lists created by this method can offer labelled training sets, which is an essential prerequisite for the future use of machine-learning-based detection methods. Two URL lists are normally produced by list-based phishing website detection methods, i.e., a blacklist and a whitelist. Figure 4 shows the general mechanism of list-based phishing URL detection methods. The antiphishing companies use the reports from the community to create one blacklist and one whitelist. The computing devices use these two lists to detect malicious websites. The whitelist contains the user-trusted URLs. In contrast, when one URL is on the blacklist, it is recognised as a malicious URL. However, with the list-based method there remains an ongoing challenge of the detection of unknown URLs. It is difficult to classify an unknown URL that is not on any list. The common policy is to recognise that as a benign URL. If a new malicious website uses this unknown URL, the false negative could potentially harm users. Attackers take advantage of this loophole and keep changing URLs for their phishing websites to ensure the new URLs are not on the blacklist.

**Figure 5:**
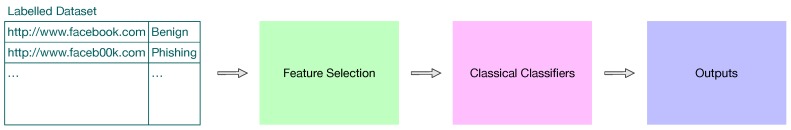
The general process of a classical supervised learning-based malicious website detection method.

Phishing website detection is modelled as a supervised machine learning problem. Components from websites, such as URL, HTML, etc., are used as the training data for building a model to conduct malicious website detection. Classifiers play a vital role in supervised machine learning methods. There are several classical and popular supervised machine learning algorithms, such as kNN, SVM, etc. that have already been used for malicious website detection applications. Figure 5 shows the general process of a classical supervised learning-based malicious website detection method. The feature selection process is an initial and essential step for these classical classifiers. Informative features can help improve the detection performance, but excellent feature selection needs the expert knowledge from a cyber security perspective. Furthermore, it is always difficult to decide the best features for a particular application. Feature selection may cause a drastic loss of valuable information.

**Figure 6:**
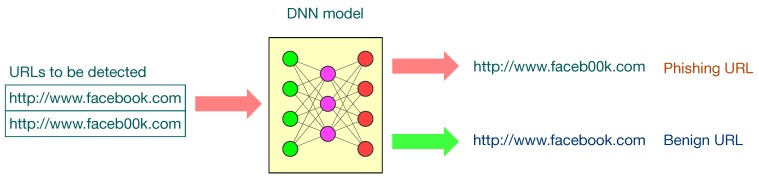
Deep-learning-based malicious website detection.

Recently, the use of deep learning has improved the performance of many applications in image processing [20], computer vision [21], acoustic classification [22], natural language processing [23], etc. Many research works also utilise deep learning techniques in malicious website detection. Additional to the significant performance improvement, deep learning has the advantage of being featureless. As shown in Figure 6, the deep-learning-based methods do not require feature selection. The unprocessed data could be used to train a model without any extra effort, and deep learning algorithms will help select the best patterns for the final decision. Motivated by these facts, this paper also designs a deep learning model and uses unprocessed URLs to derive a deep-learning-based light-weight phishing detection sensor for inference.

To enable the light-weight phishing detection sensor, another question this paper would like to address in this paper is “Can the proposed deep learning model be integrated into a resource-constrained computing device?” The paper aims to design a phishing detection sensor to achieve accurate and efficient phishing detection. By applying the designed system, it is not necessary to install antiphishing software on every single computing device and Internet of Things (IoT) device. Only the designed sensor is required for one household or office between the devices and the router. The proposed model can also be implemented into the router directly due to its computational efficiency. To summarise, this paper implements a phishing detection prototype sensor with the integration of the proposed deep learning methods.

**Figure 7:**
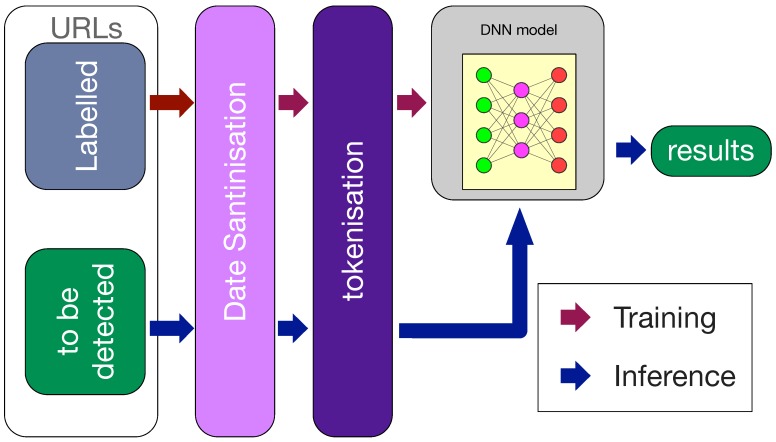
System structure.

This section will give the details of the proposed deep-learning-based phishing URL detection method. Figure 7 shows an overview of the proposed method.

## 4. Method

The first step of the proposed method is data sanitisation. In this step, the common URL prefixes, such as http://, https:// and www, are deleted to prevent the impact of URL presentations of the different datasets on phishing URL recognition performance. Without pruning prefixes, the inconsistency of URL formats can easily affect the quality of the model. For example, all the URLs in some phishing URL datasets contain the http prefix, which means that the trained model will falsely classify all of the URLs with the http prefix as phishing. The shorter representation will also accelerate the inference, which is also a main considerable factor for resource-constrained devices.

The tokeniser is used to vectorise each character in URLs. Character-level tokenisation is used instead of word-level analysis because URLs usually use words without any meaning. More information is contained at the character level. The attackers also mimic the URLs of authentic websites by changing several characters that are not noticeable. For example, they may change facebook.com to faceb00k.com, replacing ”oo” with ”00”. The character-level tokenisation helps find this mimic information, improving the performance of malicious URL detection.

**Figure 8:**
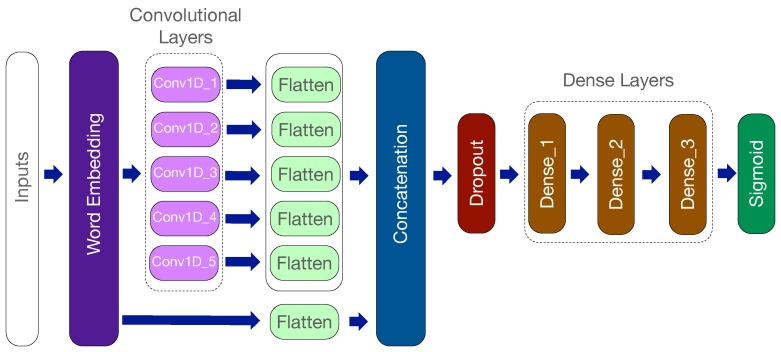
Structure of the proposed DNN model.

This paper proposes an innovative deep neural network for malicious URL detection. As shown in Figure 8, the proposed Deep Neural Network (DNN) model has the following layers: (1) embedding layers; (2) convolutional layers; (3) concatenation layer (4) dropout layers; (5) dense layers; (6) sigmoid layers. Table 1 shows the configuration of the layers of the proposed deep-layered model. The output dimension of the word embedding layer, the number of filters, and the kernel size of the convolutional layers, the rate of the dropout layer and the number of units of the dense layers are shown. Here are the details for each type of layer in the configuration.

**Figure 9:**
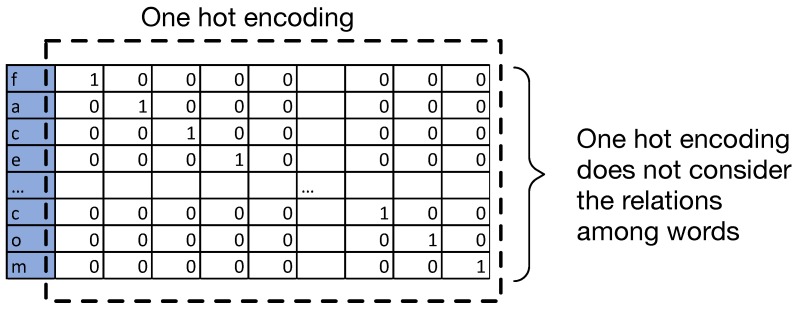
Example of one hot encoding.

**Figure 10:**
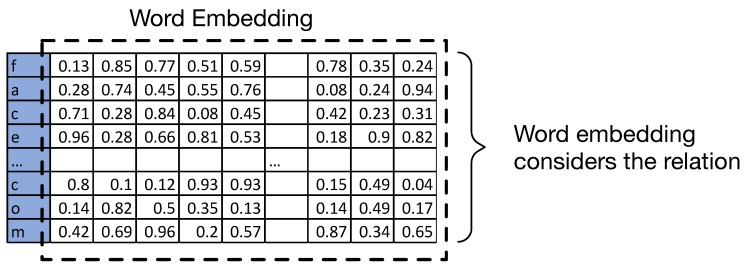
Example of word embedding.

**Embedding layer:** The embedding layer is usually used in the first layer of the DNN structure for a Natural Language Processing(NLP) problem. Additional to the tokenisation, the embedding layer will return a vector. Figure 9 and Figure 10 show examples of the simple one hot encoding and the used word embedding. Different from one hot word using binary representations for each word, the coefficients in the vector returned from the embedding layer are able to indicate the relations among characters, which can help improve the performance of NLP-related research questions. The proposed network uses embedding word configuration.

**Convolutional layers:** Following the embedding layer, five convolutional layers are used. For each convolutional layer, the kernel, a.k.a. a convolutional filter, is applied to extract the most useful features and remove unnecessary information. The element-wise multiplication and the summary operations occur between the filter and the relevant part of data, and the filter slides through the data to generate the features. Instead of a common sequential structure of convolutional neural networks, parallel convolutional layers are used. Each layer considers one window size of consecutive characters and extracts features from them. The rectified linear unit (ReLU) activation function is also used following each convolutional layer. The output from each convolutional layer is then flattened and subsequently concatenated.

**Concatenation layer:** This layer is used to concatenate the features from previous layers for further processing. Different from simply concatenating the outputs from convolutional layers, the output from the embedding layers are also combined. In addition, the output from the embedding layer (without the convolutional filtering) preserves the original information of content that can be used to detect malicious URLs as well.

**Dropout layer:** Dropout layer is a regularisation technique that is used to prevent overfitting during the training phase [24]. Neurons are randomly selected and ignored by the dropout layer during the training phase. Those ignored neurons are temporally removed on the forward pass, and their weights are not updated on the backward pass.

**Dense layers:** A dense layer is a fully connected feedback layer that equips the proposed model with the more capabilities for extracting the informative features. Following the dropout layer, three dense layers are used to analyse the patterns from the concatenation layer. One ReLU activation function also follows each dense layer.

**Sigmoid layer:** The sigmoid function is used in this layer to determine the malicious URLs. The range of the output from a sigmoid function is between 0 and 1, which is used in the final layer of the proposed model to show the prediction probability.

## 5. Evaluation

This section discusses the performance of the proposed model. As discussed, the configuration of the proposed model is shown in Table 1. A PC with a Graphics Processing Unit (GPU) is used to train and evaluate the model. The computer used has an Intel Core i7 8 core CPU 3.60 GHz processor, 16 GB memory, and Nvidia GeForce GTX 1060 6 GB GPU. A total of 1,523,966 URLs were used, where 999,996 were legitimate URLs and 523,970 were phishing URLs. The legitimate URLs are from the list of Alexa top 1 million sites [25], hphosts [5], Joewein [4], malwaredomains [26], and phishtank [3]. Before using them, repeated URLs were removed to construct a dataset. The dataset was randomly split into a training set and a test set. The percentage of testing instances was 10%. The true detection rate was used as the accuracy metric, i.e., the ratio between the number of correct detected instances and the total number of instances.

Using the proposed model can achieve an 86.630% true detection rate. Many similar deep-learning-based URL detection models use similar structures but configurations with different numbers of dense layers and convolutional layers. Therefore, in the following, the effect of the dense layers, convolutional layers, and concatenation of the output from the embedding layer will be discussed.

First, the effect of the dense layers is shown in Table 2. It is expected that increasing the number of dense layers can improve performance, so we first investigate the effect of the number of dense layers. The proposed model has 4 dense layers (3 dense layers plus the sigmoid layer). Table 2 shows that the true detection rate gradually increases with the increasing number of dense layers. The proposed method can achieve an 86.630% true detection rate. With 1, 2, and 3 dense layers, the true detection rates are 86.537%, 86.538%, and 86.542%, respectively.

Table 3 shows the effect of convolutional layers. A similar observation was also found here. Th increasing number of convolutional layers can help improve the performance the URL-based phishing detection. When using 1, 2, 3, and 4 convolutional layers, the deep learning model can achieve true detection rates of 85.401%, 85.832%, 86.169%, and 86.439%, respectively. The true detection rate of the proposed method is highest at 86.630%.

In this paper, we also propose to concatenate the output from the word embedding layer to enable the dense layers to have the unprocessed information as well. Performance improvement can also be found using this strategy, as shown in Table 4. Without the concatenation of the output from the embedding layer, the true detection rate drops from 86.630% to 83.472%.

## 6. Prototype Implementation

The proposed method is implemented by integrating the proposed deep-learning-based method into resource-constrained devices. In this work, Raspberry Pi 3 B+ was chosen to implement our prototype. Raspberry Pi 3 B+ has a Quad core 1.4 GHz 64 bit CPU with 1 GB RAM. It is powered by 5 V power input or battery and has various Input/Output (IO) ports, such as 4 USB 2.0 ports, 40-pin general-purpose input/output (GPIO) header, and Camera Serial Interface (CSI) port. Raspberry Pi 3 B+ also supports the common network ports, such as 2.4 GHz and 5 GHz IEEE 802.11 wireless cards and the Ethernet. The abundance of network cards makes Raspberry Pi 3 B+ a good candidate for simulating a router.

**Figure 11:**
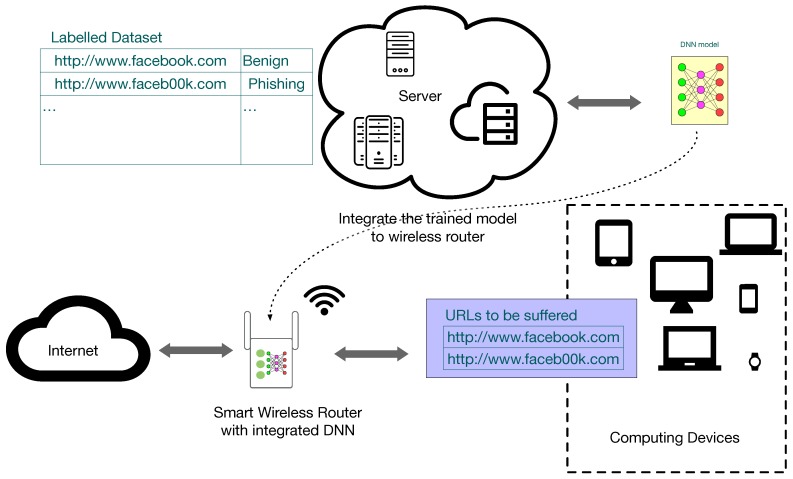
The implementation process.

Figure 11 shows the implementation process for the whole system. We first use the labelled URLs to train the DNN model by using powerful computing devices, such as GPU servers, rack servers, or cloud servers. The trained DNN model is then transferred to the intelligent WiFi router that acts as the phishing malicious URL sensor. When the intelligent WiFi router receives URL requests, it conducts phishing detection by using the integrated DNN model before requiring a domain name system (DNS) server. When the URL is recognised as malicious, the smart WiFi router will raise an alarm to the user and block the user’s access to that URL.

To evaluate the efficiency of the proposed model, we measure the computational time of each step of the proposed method using Raspberry Pi. Table 5 demonstrates the execution time of data sanitisation, tokenisation, and inference using DNN. A total of 10 trials were executed, and we calculated the mean execution time for each step. The inference costs 105 ms for each URL request, which occupies most of the running time. In the meantime, the data sanitation and tokenisation take no more than 1 ms. Totally, the proposed phishing detection method uses approximately 110 ms to evaluate each URL request, which can enable real-time malicious detection.

The word-level word embedding method along with character-level word embedding is used in [10]. To compare that work and show the efficiency of the proposed model, a deep learning model is implemented using both word-level and character-level word embedding methods. The same convolutional layers (as shown in Figure 8) are applied on the outputs of those two word embedding layers. Two outputs from convolutional layers are concatenated for further processing by dropout, dense, and sigmoid layers to make a decision. This deep learning model was evaluated in Raspberry Pi, but an out-of-memory (OOM) error occurred when running it. In other words, there was not sufficient memory in the resource-constrained Raspberry Pi to execute the implemented deep learning model with both word-level and character-level word embedding methods. This further confirms the efficiency of the proposed light-weight model. To compare the performances, the proposed model and the model with both word-level and character-level word embedding methods are executed in the PC. The execution time of DNN inference with the proposed model is 67 ms, while the model with both word-level and character-level word embedding methods needs 96 ms. The execution time significantly reduces by 30% using the proposed model.

## 7. Conclusions

This paper proposed a multispatial convolutional neural network to enable an accurate and efficient phishing detection sensor. Extensive evaluations were conducted to show the performance of the proposed method. The true detection rate of the proposed method can achieve 86.63%. A prototype by using Raspberry Pi was also implemented to enable real-time phishing URL detection. With the proposed method, the execution time reduces by 30%, and real-time detection is realised in a resource-constrained device.

In the future, a webpage-content-based phishing detection model using deep learning can be proposed and implemented in a resource-constrained sensor as well.

## Figures and Tables

**Table 1:** Architecture configuration of the proposed DNN model.

	Output Dimension	
Word Embedding	32	
	Number of Filters	Kernel Size
Conv1D_1	256	2
Conv1D_2	256	3
Conv1D_3	256	4
Conv1D_4	256	5
Conv1D_5	256	10
	Dropout Rate	
Dropout	0.5	
	Number of Units	
Dense_1	128	
Dense_2	128	
Dense_3	128	

**Table 2:** The effect of the dense layers.

	Accuracy
Proposed	**86.630%**
1 Dense Layer	86.537%
2 Dense Layers	86.538%
3 Dense Layers	86.542%

**Table 3:** The effect of the convolutional layers.

	Accuracy
Proposed	**86.630%**
1 Convolutional Layer	85.401%
2 Convolutional Layers	85.832%
3 Convolutional Layers	86.169%
4 Convolutional Layers	86.439%

**Table 4:** The effect of the concatenation of the output from the embedding layer.

	Accuracy
Proposed	**86.630%**
No Concatenation	83.472%

**Table 5:** Execution time in the prototype.

	Execution Time (ms)
Data Sanitisation	0.0106
Tokenisation	0.1997
DNN Inference	105

## Abbreviations

The following abbreviations are used in this manuscript:

URL	Uniform resource locator
APWG	Anti-phishing working group
FBI	Federal Bureau of Investigation
SVM	Support vector machine
kNN	k-nearest neighbours algorithm
CNN	Convolutional neural network
ReLU	Rectified linear unit
DNS	Domain name system
